# Coupling of Transcription and Translation in Archaea: Cues From the Bacterial World

**DOI:** 10.3389/fmicb.2021.661827

**Published:** 2021-04-29

**Authors:** Albert Weixlbaumer, Felix Grünberger, Finn Werner, Dina Grohmann

**Affiliations:** ^1^Department of Integrated Structural Biology, Institut de Génétique et de Biologie Moléculaire et Cellulaire (IGBMC), Illkirch, France; ^2^Université de Strasbourg, Strasbourg, France; ^3^CNRS UMR7104, Illkirch, France; ^4^INSERM U1258, Illkirch, France; ^5^Institute of Microbiology and Archaea Centre, University of Regensburg, Regensburg, Germany; ^6^RNAP Lab, Division of Biosciences, Institute for Structural and Molecular Biology, London, United Kingdom; ^7^Regensburg Center for Biochemistry, University of Regensburg, Regensburg, Germany

**Keywords:** RNA polymerase, ribosome, archaea, expressome, Spt4/5, NusG, Nus

## Abstract

The lack of a nucleus is the defining cellular feature of bacteria and archaea. Consequently, transcription and translation are occurring in the same compartment, proceed simultaneously and likely in a coupled fashion. Recent cryo-electron microscopy (cryo-EM) and tomography data, also combined with crosslinking-mass spectrometry experiments, have uncovered detailed structural features of the coupling between a transcribing bacterial RNA polymerase (RNAP) and the trailing translating ribosome in *Escherichia coli* and *Mycoplasma pneumoniae*. Formation of this supercomplex, called expressome, is mediated by physical interactions between the RNAP-bound transcription elongation factors NusG and/or NusA and the ribosomal proteins including uS10. Based on the structural conservation of the RNAP core enzyme, the ribosome, and the universally conserved elongation factors Spt5 (NusG) and NusA, we discuss requirements and functional implications of transcription-translation coupling in archaea. We furthermore consider additional RNA-mediated and co-transcriptional processes that potentially influence expressome formation in archaea.

## Introduction

The controlled and coordinated expression of genes plays a fundamental role in all cellular life forms and occurs in two steps: transcription of DNA to RNA by RNA polymerase (RNAP) and translation of RNA to protein by the ribosome. Cellular RNAPs share a conserved core architecture (Hirata et al., 2008; Korkhin et al., 2009; Werner and Grohmann, 2011; Jun et al., 2014; Griesenbeck et al., 2017). However, the archaeal RNAP structure, subunit composition, and use of basal transcription factors (TF) are more closely related to eukaryotic RNAP II than the bacterial counterpart. Ribosomes are large ribonucleoprotein particles that consist of two subunits that entail ribosomal proteins (r-proteins) and rRNAs. While the general organization and function of the ribosome is universally conserved, the complexity and protein content of ribosomes increases from bacteria to archaea to eukaryotes (Armache et al., 2013; Yusupova and Yusupov, 2014; Ferreira-Cerca, 2017). In fact, differences in the transcriptional and translational apparatus reflect the increase in complexity during evolution (Armache et al., 2013). For example, major differences in ribosome subunit composition are already apparent in the four phylogenetically distinct (super-)phyla: Thaumarchaeota, Aigarchaeota, Crenarchaeota, and Korarchaeota (TACK); Euryachaeota; Diapherotrites, Parvarchaeota, Aenigmarchaeota, Nanoarchaeota, and Nanohaloarchaeota (DPANN); and Asgard archaea.

Prokaryotes lack a nucleus, so transcription and translation occur in the same cellular compartment, the cytoplasm. Biochemical evidence and electron micrographs of lysed bacteria led to the early proposal and realization that translation occurs co-transcriptionally (Byrne et al., 1964; Miller et al., 1970). This prompted the question whether coordination or coupling of elongating RNAP with the pioneering ribosome mutually influences transcription and translation. Data from bacteria provided direct evidence that rates of transcription and translation are interdependent, at least in some species and for some transcription units (Landick et al., 1985; Proshkin et al., 2010; Castro-Roa and Zenkin, 2012; Zhu et al., 2019; Johnson et al., 2020; Stevenson-Jones et al., 2020). However, recent work in *Bacillus subtilis* showed that coupling of transcription and translation is not conserved across all bacteria (Johnson et al., 2020). Recently, single-particle cryo-electron microscopy (cryo-EM) and cryo-electron tomography (cryo-ET) was used to elucidate structural details of the coupled bacterial RNAP and ribosome, a macromolecular assembly termed “expressome.” It highlighted roles of transcription elongation factors NusG and/or NusA that physically connect RNAP with the ribosome (Demo et al., 2017; Kohler et al., 2017; O’Reilly et al., 2020; Wang et al., 2020; Webster et al., 2020).

In contrast, little is known about the coupling of transcription and translation in archaea. It is unclear if direct interactions between RNAP and ribosomes occur or if their association is solely mediated by the shared mRNA. Likewise, the contribution and regulatory role of accessory transcription factors is unknown (McGary and Nudler, 2013; Artsimovitch, 2018). Based on the structural information of the bacterial expressome(s), we discuss whether a coupling between the archaeal RNAP and ribosome can take place in a comparable manner. While molecular structures often guide hypotheses about underlying molecular mechanisms, they rarely suffice to provide the complete picture. We discuss how additional functional evidence obtained *in vivo*, including reporter gene assays and systems biology data such as transcriptome analyses and ribosome profiling, can shed light on the coupled gene expression in archaea. Finally, gene expression takes place in the context of other essential physiological processes. Hence, events like RNA processing and degradation, and their impact on transcription, translation, and their coupling are important to consider.

## Structural Insights Into the Bacterial Expressome

**Figure 1:**
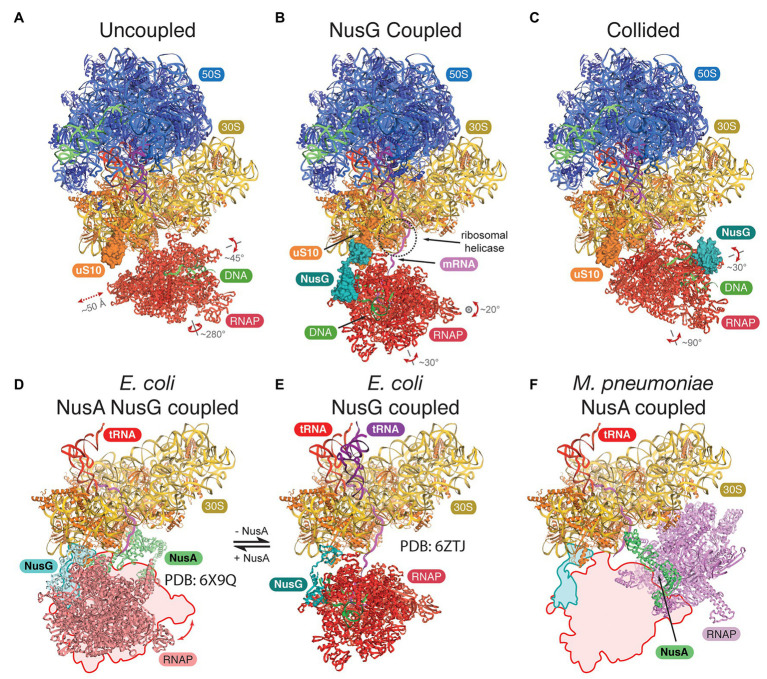
Structures of the bacterial expressomes. **(A)** At mRNA spacings separating the RNA polymerase (RNAP) active site by more than ~35 nucleotides from the ribosomal P-site, and in absence of any coupling factor, RNAP adopts a wide range of orientations relative to the ribosome (uncoupled state; compare extent of translations and rotations). **(B)** Addition of the coupling factor NusG (turquoise) results in the formation of a physical link between RNAP and the ribosome (through uS10, orange) and restrains the rotational freedom. The emerging mRNA transcript (magenta) aligns with the ribosomal helicase (r-proteins uS3 and uS4, dashed circle; NusG coupled state). **(C)** Once the ribosome approaches RNAP further (mRNA spacings less than ~34 nucleotides), the expressome adopts a conformation similar to a previously observed collided state, where NusG can no longer form a physical bridge (collided state). **(D–F)** Comparison of transcription factor coupled states in *Escherichia coli* and *Mycoplasma pneumoniae*. The NusG-coupled state **(E)** gets stabilized by NusA in a similar relative orientation [**D**, compare outline with model, differences are within rotational freedom indicated in panel **(A)**]. In contrast, the NusA-coupled state observed in *M. pneumoniae* requires a major change in position and orientation of RNAP **(F)**, compare outline and model, *E. coli* RNAP and ribosome were docked into deposited cryo-electron tomography (cryo-ET) map (O’Reilly et al., 2020).

Attempts to gain structural insights into bacterial expressomes were based on two approaches: (i) cryo-EM of samples formed by direct reconstitution of purified *Escherichia coli* components on mRNA substrates, which direct a precise spacing between RNAP and the 70S ribosome (Wang et al., 2020; Webster et al., 2020), or (ii) direct visualization using cryo-ET in combination with in-cell cross-linking mass spectrometry in *Mycoplasma pneumoniae* (O’Reilly et al., 2020). With sufficient mRNA separating the two machineries, RNAP adopts a wide range of orientations, the assembly is highly flexible, and the mRNA is the only consistent connection (Figure 1A). In *E. coli*, adding NusG restrains RNAP and aligns the mRNA with the ribosomal helicase (Figure 1B), proposed to prevent secondary structure formation in the transcript (Webster et al., 2020). Addition of the TF NusA stabilizes the NusG-coupled expressome (Wang et al., 2020; Figures 1D,E). In contrast, in *M. pneumoniae*, NusA alone appears to couple the two machineries without a role for NusG, albeit in a different relative orientation (O’Reilly et al., 2020; Figure 1F). This is consistent with the weak sequence conservation in the NusG KOW domain of *E. coli* and *M. pneumoniae* and suggests that a different mechanism for coupling evolved in this minimal genome species.

All three studies concluded that short spacings between RNAP and the ribosome, either directed by the mRNA or by adding a drug to halt RNAP, form expressomes that resemble an earlier lower-resolution reconstruction formed by collision of a translating ribosome with a stalled RNAP (Figure 1C; Kohler et al., 2017). Importantly, while RNAP is still mobile in this collided conformation, NusG cannot simultaneously bind RNAP and the ribosome and therefore cannot form a physical link.

While it is tempting to suggest uncoupled, NusG coupled, and collided expressome structures represent a ribosome approaching RNAP (in agreement with a reduction in RNA separating the two machineries), there is no other experimental evidence to support this chronological order of events, and this remains subject for further research.

## Is Transcription Coupled to Translation in Archaea?

For the archaeon *Thermococcus kodakarensis*, DNA-attached polysomes have been visualized by electron microscopy (French et al., 2007) suggesting that transcription translation coupling (TTC) occurs in archaea. Given the bacterial expressome, the question arises whether the archaeal machineries are compatible with this architecture. To answer this question, the bacterial transcription and translation apparatus has to be compared concerning (i) the overall RNAP architecture, (ii) the RNA length bridging the RNAP active site with the ribosomal P-site (carrying the peptidyl-tRNA), (iii) the presence of NusG or NusA-like factors, and (iv) the conservation of interaction surfaces.

In contrast to bacterial RNAPs, archaeal-eukaryotic RNAPs contain subunits Rpo4/7 (the stalk domain), which binds nascent RNA (Todone et al., 2001; Meka et al., 2005) and stimulate RNAP processivity (Hirtreiter et al., 2009) suggesting the stalk guides the RNA away from RNAP once it emerges from the RNA exit channel. Complexes between bacterial RNAP and 70S ribosomes could be observed for RNA spacers as short as 29 nt separating the RNAP active site from the ribosomal P-site (Wang et al., 2020). However, NusG-mediated coupling appears to be compatible only with spacer lengths greater than at least ~34 nt (Webster et al., 2020). Cryo-EM reconstructions (Bernecky et al., 2016; Ehara et al., 2017) and single-molecule FRET studies (Andrecka et al., 2008) of eukaryotic elongation complexes showed that transcripts of 14–29 nt reach the stalk base. Longer RNAs could not be mapped and appeared to be flexible. This suggests the attachment of longer RNAs to the stalk is transient or they are no longer associated with the stalk. In the context of the archaeal RNAP and assuming that the nascent RNA binds the stalk, for TTC to occur, a longer mRNA segment is required that can traverse the stalk before being fed into the ribosome in contrast to the bacterial situation. Alternatively, the mRNA might be detached from Rpo4/7 and directly enter the ribosome.

**Figure 2:**
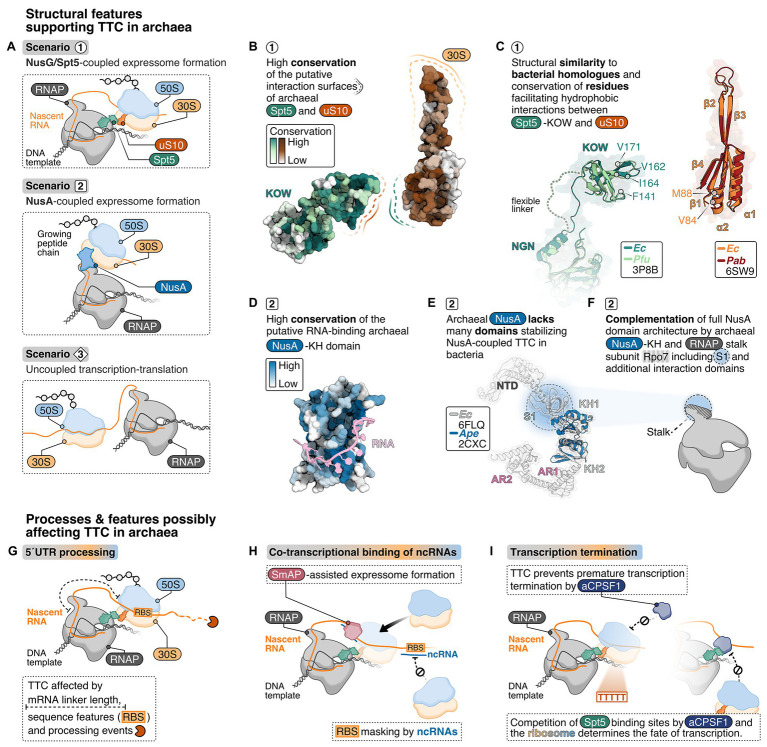
Structural criteria and cellular processes that might mediate and influence transcription-translation coupling in archaea. **(A)** Possible scenarios for transcription translation coupling (TTC) in archaea derived from structural insights into expressome formation in bacteria: NusG/Spt5-coupled expressome formation is a possible scenario 1 representing the archaeal equivalent to the NusG-coupled state in *E. coli* (Webster et al., 2020). In addition, one could also imagine NusA-coupling (scenario 2), similar to what has been observed in *M. pneumoniae* (O’Reilly et al., 2020) or no coupling at all (scenario 3). **(B)** Analysis of conserved regions in archaeal Spt5 and uS10 using ConSurf (Ashkenazy et al., 2016). About 100 archaeal Spt5 and uS10 sequences were aligned, and their conservation score projected color-coded from white (0, not conserved) to dark green or dark-red (9, highly conserved), respectively, on the surface of *Pyrococcus furiosus* Spt5 and *Pyrococcus abyssi* uS10 structure. **(C)** Superimposition of archaeal and bacterial Spt5/NusG and (bacteria: dark-green, PDB: 6ZTJ, *E. coli*; archaea: light-green, PDB: 3P8B, *P. furiosus*) and uS10 (bacteria: orange, PDB: 6ZTJ, *E. coli*; archaea: red, PDB: 6SW9, *P. abyssi*). Residues important for the interaction with NusG are highlighted. **(D)** Conservation analysis of archaeal NusA proteins. Conservation scores from white (0, not conserved) to dark-blue (0, highly conserved) were calculated based on the comparison of archaeal NusA proteins and projected on the surface of *Aeropyrum pernix* NusA. A superimposed model of bacterial RNA (Beuth et al., 2005) is shown in pink. **(E)** The *E. coli* NusA structure (gray, PDB: 6FLQ) overlayed with *A. pernix* NusA (Shibata et al., 2007) (blue, PDB: 2CXC). **(F)** Cartoon depiction of the archaeal RNAP highlighting the hypothesis that the S1 domain of the stalk-forming subunit Rpo7 and the archaeal NusA form a homologue of bacterial NusA. TTC in archaea may be affected by co-transcriptional processes and features depicted in **(G**–**I)**, including 5'UTR length and processing **(G)**, co-transcriptional binding of non-coding RNAs (ncRNAs) **(H)** and the transcription termination pathway **(I)**.

In the *E. coli* expressome, RNA-dependent TTC is further mediated by NusG, which is the only universally conserved TF (Werner, 2012). In archaea and eukaryotes, the NusG homolog is called Spt5 and forms a heterodimer with Spt4. NusG/Spt5 has an N-terminal NGN domain and a C-terminal KOW domain, which bind the RNAP clamp domain and the r-protein uS10, respectively (Figure 2A). At the majority of genes, archaeal Spt4/5 associates with the elongation complex proximal to the promoter and reflects the RNAP association pattern (Smollett et al., 2017). This suggests early Spt4/5 recruitment to RNAP even for short transcripts and thus a coupling function may also occur early in transcription. NusG/Spt5 are structurally conserved (Figures 2B,C; Hirtreiter et al., 2010; Martinez-Rucobo et al., 2011; Liu and Steitz, 2017). Hence, the interaction interfaces between NusG/Spt5 and RNAP and/or the ribosome might also be conserved.

First, we focus on the NusG-mediated contact between RNAP and ribosome because biochemical data suggest this to be the prevalent arrangement of the expressome *in vivo* (Saxena et al., 2018; Washburn et al., 2020). The binding site of NusG/Spt5 on RNAP is conserved according to structural data in all three kingdoms of life (Figures 2B,C; Klein et al., 2011; Martinez-Rucobo et al., 2011; Ehara et al., 2017; Kang et al., 2018). While structural data on archaeal Spt5 (aSpt5) interacting with the archaeal ribosome are missing, the length and mobility of the linker connecting the NGN and KOW domain in aSpt5 resembles NusG. Thus, a similar interaction as observed for the bacterial NusG-coupled expressome is feasible. Furthermore, bacterial RNAP exhibits substantial rotational and translational freedom with respect to the ribosome even in the NusG-coupled expressome. Modeling of an archaeal expressome based on bacterial RNAP orientations (Webster et al., 2020) shows that most orientations would require a different stalk orientation to avoid steric overlap with the 30S subunit (Coureux et al., 2020). Archaeal RNAP might either be more restricted in its orientation relative to the ribosome or adopt different orientations compatible with the stalk that have been modeled to be possible without steric clashes between RNAP and the ribosome (Kohler et al., 2017).

Bacterial uS10 provides a hydrophobic pocket for the KOW domain of NusG to insert several hydrophobic residues (Webster et al., 2020). Residues V84 and M88 in uS10 form one edge of the hydrophobic pocket in close proximity to F141, F144, and I164 in the NusG-KOW domain (Burmann et al., 2010; Webster et al., 2020). V84 and M88 in uS10 and F141 and I164 in the KOW domain are conserved among bacterial and archaeal proteins (Figures 2B,C; Melnikov et al., 2018) suggesting the hydrophobic interaction between NusG/Spt5-KOW and uS10 might be conserved. Moreover, the structure of bacterial and archaeal uS10 is conserved (Figure 2C) and residues in the putative interaction surface (β-strand 1 and 4, α-helix 2) of archaeal uS10 with Spt5 are highly conserved among archaeal uS10 proteins suggesting that the amino acid identity might play a role for the function and interaction of archaeal uS10 (Coureux et al., 2020; Figure 2B). Despite the conserved phenylalanine residues and overall sequence conservation of aSpt5, organisms of the euryarchaeal and crenarchaeal phylum do not share a high sequence conservation with bacterial KOW sequences.

The interactions in the expressome are not conserved across all bacteria and alternative coupling mechanisms have evolved. In *M. pneumoniae*, the bacterial elongation and termination factor NusA couples RNAP and the ribosome (O’Reilly et al., 2020). Commonly, bacterial NusA proteins contain an N-terminal domain (binds RNAP), and a S1 and two KH domains (bind RNA). *Mycoplasma pneumoniae* NusA contains an additional flexible C-terminal extension not found in *E. coli* or *B. subtilis*, which contacts multiple r-proteins on the ribosome (Figure 1D). Consequently, the relative orientation of the ribosome to RNAP differs significantly from the *E. coli* expressome architecture (Figure 1D). NusA is also able to stabilize NusG-coupled expressomes in *E. coli* mediated by one of the KH domains (Wang et al., 2020). NusA-like homologs can be found in all archaeal phyla indicating a widespread distribution of this transcription factor but its function is unclear (Shibata et al., 2007). The domain organization differs significantly from bacterial NusA because archaeal NusA (aNusA) only contains KH domains but lacks the NTD, S1 domain and C-terminal extension that interacts with the ribosome in *M. pneumoniae*. Nevertheless, the structure of the bacterial and archaeal KH domains in NusA are highly conserved (Figures 2D,E) and aNusA also binds RNA (Shibata et al., 2007). It has been suggested that the RNAP interaction platform and S1 domain of Rpo7 in conjunction with the two KH domains of aNusA form the domain complement of bacterial NusA (Figure 2F; Belogurov and Artsimovitch, 2015; Fouqueau et al., 2018).

It is noteworthy that the archaeal domain of life encompasses highly diverse organisms, of which only a few model organisms have been studied so far. As documented for the bacterial world (Irastortza-Olaziregi and Amster-Choder, 2020; Johnson et al., 2020), expressome formation might occur in some archaeal species but not in others.

## Co-Transcriptional Processes and Transcriptomic Features Affecting Transcription-Translation Coupling in Archaea

The expressome structures illustrate the highly coordinated interplay of two molecular machineries. However, the expressome is not an isolated complex but operates with high specificity in a crowded cytoplasm where myriads of molecular processes occur simultaneously. In archaea, a number of transcriptional and co-transcriptional steps have been identified that might prevent the immediate loading of the ribosome onto the mRNA. Among others, processes like co-transcriptional RNA processing, binding of non-coding RNAs (ncRNAs) to and association of RNA chaperones and transcription termination factors with the RNA may influence expressome formation and will be shortly discussed in this section (compare Figures 2G–I).

Coupling of the ribosome to RNAP requires the mRNA to span the distance between the RNAP active site and the ribosomal P-site to provide enough space for both machineries (Figure 2G). Typically, regulatory sequences that confer translation initiation are encoded in the 5' untranslated region (5'-UTR). Some archaeal mRNAs have a short 5' UTR or none at all (analyzed for *Haloferax volcanii*, *Thermococcus onnurineus*, *Pyrococcus abyssi*, *Saccharolobus solfataricus*, Heyer et al., 2012; Xu et al., 2012; Beck and Moll, 2018). The relative number of leaderless mRNAs ranges between 1.4 and 72%. The mechanism of mRNA recognition and ribosome association appears to be highly diverse in prokaryotes, and we do not know whether the initiation mechanism influences and correlates with expressome formation (Wen et al., 2020). mRNAs that lack a ribosomal binding site (RBS) can also emerge from RNA processing events at the 5'-end that lead to cleavage of the 5'-UTR (Qi et al., 2017; Figure 2G). As shown for several bacterial (Mäder et al., 2004; Ramirez-Peña et al., 2010; Lioliou et al., 2012) and for the archaeal organisms *Methanocaldococcus jannaschii* and *Marinobacter psychrophilus* (Zhang and Olsen, 2009; Qi et al., 2017), processing of the mRNAs can stabilize transcripts and regulate translation of r-proteins (Qi et al., 2017) and mRNAs from multicistronic operons. In this case, the timing of mRNA processing and translation seems important to avoid conflicts between these two processes.

Co-transcriptional binding of a small regulatory ncRNA to an mRNA is a common posttranscriptional regulation mechanism in prokaryotes that influences RNA stability and translational efficiency of mRNAs in response to changing environmental conditions (Babski et al., 2014; Hör et al., 2018). For *H. volcanii* and *Methanosarcina mazei* small ncRNAs have been detected that can potentially bind to the 5' UTR thereby potentially masking the RBS (Jäger et al., 2009; Soppa et al., 2009; Heyer et al., 2012; Gelsinger and DiRuggiero, 2018; Figure 2H). For example, the small RNA_41_ in *M. mazei* binds multiple RBS in a polycistronic mRNA and decouples transcription and translation (Buddeweg et al., 2018).

In bacteria, ncRNA-mRNA hybridization is often mediated by the RNA chaperone Hfq, which belongs to the Sm protein family (Vogel and Luisi, 2011). Hfq can bind RNA co-transcriptionally (Kambara et al., 2018) and plays a role in transcription termination/antitermination (Rabhi et al., 2011; Sedlyarova et al., 2016), ribosome biogenesis (Andrade et al., 2018) and ribosome association with the mRNA in bacteria (Chen et al., 2019). In archaea, a *bona fide* Hfq protein is rarely encoded. More often, single or multiple genes encode an archaeal Sm-like protein (SmAP; Reichelt et al., 2018). Similar to bacterial Hfq, archaeal SmAPs were shown to bind RNAs (Nielsen et al., 2007; Fischer et al., 2010; Märtens et al., 2015). Hence, co-transcriptional association of a ncRNA at the 5' UTR (potentially supported by a SmAP) would prevent ribosome association with the 5' UTR (Figure 2H). Co-immunoprecipitation experiments showed that SmAPs not only bind RNAs but also r-proteins (Fischer et al., 2010). It is conceivable that SmAPs participate in posttranscriptional regulation, translation, or act as a bridging factor to recruit ribosomes to the mRNA (Figure 2H).

Lastly, the transcription termination pathway might be decisive whether TTC can occur, or vice versa (Figure 2I). In archaea, transcription terminates *via* two mechanisms that are not necessarily mutually exclusive: (i) intrinsic termination at poly(U) stretches (Santangelo and Reeve, 2006; Hirtreiter et al., 2009, 2010; Santangelo et al., 2009; Dar et al., 2016; Berkemer et al., 2020) or (ii) factor-dependent termination assisted by the archaeal termination factor aCPSF1/FttA that binds the nascent RNA (Sanders et al., 2020; Yue et al., 2020). Importantly, aCPSF1 also enhances termination at poly(U) stretches. Termination *via* aCSPF1 involves cleavage of the transcript at the 3'-end. In *Methanococcus maripaludis* deletion of aCPSF1 resulted in altered expression levels for the majority of genes (Yue et al., 2020). Furthermore, aCPSF1-dependent termination gets stimulated by the presence of the stalk domain and Spt4/5 (Sanders et al., 2020). Even though a direct interaction between aCPSF1 and the stalk or Spt4/5 has not yet been experimentally verified, a physical interaction is likely and would be consistent with the observed increased termination efficiency. It is tempting to speculate that aCPSF1 and the ribosome interact with RNAP-bound Spt4/5 in a mutually exclusive fashion similar to Rho and the ribosome with RNAP-bound NusG in bacteria. As a consequence, transcription termination and ribosome coupling might be mutually exclusive. Ribosomes coupled to RNAP *via* Spt4/5 would prevent aCPSF1 interactions with the nascent RNA and prevent premature termination (Figure 2H). Alternatively, once aCPSF1 gains access to Spt4/5 it may interfere with TTC (Figure 2H). This would be reminiscent of the recruitment of Rho by NusG-KOW to RNAP leading to transcription termination of non-coding/untranslated RNA transcripts (Washburn et al., 2020). Whether TTC or termination prevails could be gene- or operon-specific, could be a target for regulation, and may vary from species to species. Direct, mRNA-independent interactions between the bacterial RNAP and ribosome have been shown. It is possible that in some instances, e.g., during transcription of short mRNAs, the archaeal ribosome might bind the mRNA close to the RNAP exit channel and direct contacts between the elongating RNAP and the ribosome (Wang et al., 2020; Webster et al., 2020).

## Future Perspectives

Are transcription and translation coupled in archaea similar to bacteria? We propose that this is likely, but definitive proof is still lacking. This problem can only be solved by a multidisciplinary effort that reaches beyond a molecular-structural analysis *in vitro*. In order to rationalize the underlying molecular mechanisms, we do need to understand the structural determinants of the RNAP-ribosome interactions and the potential role of general regulatory factors including NusG-Spt4/5 and NusA, as well as ribosomal proteins. A crucial question to be solved in the future is how co-transcriptional processes like SmAP binding, transcription termination, or RNA processing are coordinated with ribosome coupling in space and time. This also includes coordination of translation initiation and TTC. In bacteria, translation initiation was delineated in great detail showing that the 30S subunit is recruited to the mRNA with the help of the initiator tRNA and initiation factors before the 50S subunit joins to form the translation-competent ribosome (see for example, Milon et al., 2010; Tsai et al., 2012). In archaea, the situation is more complex as additional (eukaryotic-like) initiation factors are involved (Benelli et al., 2016). Nonetheless, even in the archaeal initiation complex, uS10 remains exposed and might be available for coupling to Spt5 (Coureux et al., 2020). Consequently, the 30S subunit is involved in translation initiation and coupling to RNAP, and it has to be seen whether these processes are compatible or mutually exclusive. To elaborate on the finer points of biologically relevant interaction networks a combination of cross-linking/mass spectrometry experiments like the recent elegant study of Rappsilber and colleagues are necessary (O’Reilly et al., 2020). Complementary to these efforts are structural biology, functional genomics, and systems biology approaches that hold great promise to ascertain (i) to which extent the coupling applies to all transcription units or whether it is limited to specific subset or classes of operons, and (ii) whether the coupling-uncoupling is a dynamic process and dependent on environmental cues and stresses, i.e., whether it is subject to regulation. Key to this approach are experiments that monitor changes in the global characteristics of transcription, such as genome-wide RNAP occupancy profiles and transcriptome analyses, in response to perturbations of translation by using ribosome inhibitors/antibiotics or ribosome variants. We have to develop high-resolution methods that combine ribo-seq/proteomics and RNAP NET-seq or ChIP-exo/transcriptomics and integrate the data to obtain a complete view of the interdependence of transcription and translation. Finally, it is important to note that archaea are evolutionary diverse and tractable archaeal model organisms are scarce. Despite the conservation of NusG, the molecular mechanisms of transcription that were revealed for Crenarchaea and Euryarchaea are distinct in many ways including the RNAP subunit composition and chromatin structure. Likewise, we only know little about the mechanisms of translation across the archaeal phyla. The properties of their ribosomes are distinct including the molecular mechanisms of translation initiation, which might have an impact on the coupling of the leading ribosome to the RNAP.

## Data Availability Statement

The original contributions presented in the study are included in the article/supplementary material, further inquiries can be directed to the corresponding author.

## Author Contributions

AW prepared the Figure 1. FG prepared the Figure 2. All authors contributed to the article and approved the submitted version.

### Conflict of Interest

The authors declare that the research was conducted in the absence of any commercial or financial relationships that could be construed as a potential conflict of interest.
